# Examining the neural correlates of error awareness in a large fMRI study

**DOI:** 10.1093/cercor/bhac077

**Published:** 2022-03-03

**Authors:** Gezelle Dali, Méadhbh Brosnan, Jeggan Tiego, Beth P Johnson, Alex Fornito, Mark A Bellgrove, Robert Hester

**Affiliations:** Melbourne School of Psychological Sciences, The University of Melbourne, Parkville, VIC 3010, Australia; Department of Experimental Psychology, University of Oxford, Oxford, OX2 6GG, UK; Oxford Centre for Human Brain Activity, Wellcome Centre for Integrative Neuroimaging, University of Oxford, Oxford, OX3 7JX, UK; The Turner Institute for Brain and Mental Health, School of Psychological Sciences, and Monash Biomedical Imaging, Monash University, Melbourne, VIC, 3800, Australia; The Turner Institute for Brain and Mental Health, School of Psychological Sciences, and Monash Biomedical Imaging, Monash University, Melbourne, VIC, 3800, Australia; The Turner Institute for Brain and Mental Health, School of Psychological Sciences, and Monash Biomedical Imaging, Monash University, Melbourne, VIC, 3800, Australia; The Turner Institute for Brain and Mental Health, School of Psychological Sciences, and Monash Biomedical Imaging, Monash University, Melbourne, VIC, 3800, Australia; The Turner Institute for Brain and Mental Health, School of Psychological Sciences, and Monash Biomedical Imaging, Monash University, Melbourne, VIC, 3800, Australia; Melbourne School of Psychological Sciences, The University of Melbourne, Parkville, VIC 3010, Australia

**Keywords:** anterior cingulate cortex, error awareness, error-monitoring processes, functional magnetic resonance imaging, insula

## Abstract

Goal-directed behavior is dependent upon the ability to detect errors and implement appropriate posterror adjustments. Accordingly, several studies have explored the neural activity underlying error-monitoring processes, identifying the insula cortex as crucial for error awareness and reporting mixed findings with respect to the anterior cingulate cortex (ACC). Variable patterns of activation have previously been attributed to insufficient statistical power. We therefore sought to clarify the neural correlates of error awareness in a large event-related functional magnetic resonance imaging (fMRI) study. Four hundred and two healthy participants undertook the error awareness task, a motor Go/No-Go response inhibition paradigm in which participants were required to indicate their awareness of commission errors. Compared to unaware errors, aware errors were accompanied by significantly greater activity in a network of regions, including the insula cortex, supramarginal gyrus (SMG), and midline structures, such as the ACC and supplementary motor area (SMA). Error awareness activity was related to indices of task performance and dimensional measures of psychopathology in selected regions, including the insula, SMG, and SMA. Taken together, we identified a robust and reliable neural network associated with error awareness.

## Introduction

Error processing facilitates goal-directed behavior through error detection and the execution of appropriate posterror adjustments. Within error processing, it is possible to delineate between errors made with and without conscious recognition. Although error processing can proceed in the absence of awareness, conscious perception of errors may subserve the implementation of remedial behaviors. Critically, deficient error awareness has been associated with symptoms of inattention, lack of insight, and perseverative behavior in several clinical conditions such as attention-deficit hyperactivity disorder (ADHD; O’Connell et al. 2009), autism spectrum disorder (ASD; Klein et al. 2013), and substance use disorder (Hester, Nestor, et al. 2009), providing impetus for investigating the underlying neurobiology of error awareness.

In performance monitoring tasks, errors are largely associated with an event-related potential (ERP) signature comprising the error-related negativity (ERN) and the error positivity (Pe). The ERN is a negative deflection with a fronto-central distribution that appears at 50–100 ms following an error (Gehring et al. 1993), whereas the Pe is a positive deflection with an approximate latency of 300–500 ms occurring over a centro-parietal location (Falkenstein et al. 1991). Neuroimaging and source localization studies have identified the anterior cingulate cortex (ACC) as the source of the ERN (Debener et al. 2005; van Veen and Carter 2006). Indeed, the ACC is consistently implicated in performance monitoring tasks and is suggested to navigate the selection and evaluation of goal-directed behaviors (Holroyd and Yeung 2012). The source of the Pe, however, remains somewhat equivocal, with reports that it arises from activity in the rostral ACC (rACC; Herrmann et al. 2004; Van Boxtel et al. 2005), prefrontal (Masina et al. 2019), and parietal cortices (van Veen and Carter 2006; O'Connell et al. 2007).

With regard to error awareness, most electrophysiological studies argue that the ERN is unaffected by conscious error perception. This pattern has been observed in a myriad of tasks, including antisaccade tasks (Nieuwenhuis et al. 2001; Endrass et al. 2007), Go/No-Go error awareness tasks (O'Connell et al. 2007; Dhar et al. 2011), and visual discrimination tasks (Steinhauser and Yeung 2010; Endrass et al. 2012). Contrastingly, the Pe has been found to be selectively modulated by error awareness such that greater amplitudes are observed following aware errors (Nieuwenhuis et al. 2001; O'Connell et al. 2007; Steinhauser and Yeung 2010; Dhar et al. 2011; Hoffmann and Beste 2015). There are, however, studies which have demonstrated that both the ERN and Pe are sensitive to error awareness (Scheffers and Coles 2000; Maier et al. 2008; Hewig et al. 2011; Wessel et al. 2011; Shalgi and Deouell 2012). Given such findings, the ERN has been proposed to be the foremost indication that an error has occurred and may serve as a feedforward input signal into systems that are more responsible for error awareness (Murphy et al. 2012; Wessel 2012), whereas the Pe reflects the accumulation of information that leads to error awareness (Klein et al. 2013).

Neuroimaging studies on error awareness have been found to accord with electrophysiological findings. Consistent with findings on the Pe, a network of frontal and parietal regions has been implicated in error awareness, namely the bilateral inferior parietal and bilateral midfrontal cortices (Hester et al. 2005; Harsay et al. 2012; Orr and Hester 2012). The insula cortex—largely the anterior insula cortex—is also widely recognized to be selectively modulated by error awareness (Klein et al. 2013). While the insula is unlikely to generate the Pe directly, it has been suggested to indirectly elicit the Pe through its functional connections with frontal and parietal cortices (Klein et al. 2007). Corroborating findings on the ERN, the relationship between awareness and the ACC remains a topic of contention. Several earlier studies that have found ACC activity to be greater for errors than correct responses have discerned no difference in the activity between aware and unaware errors (e.g. Hester et al. 2005; Klein et al. 2007). In contrast to the majority of earlier studies, recent investigations have reported dorsal ACC (dACC) sensitivity to error awareness, with increased activity observed during aware errors (Harsay et al. 2012, 2018).

Although heterogeneity in imaging modalities, sample characteristics, and study designs may contribute to discordant neuroimaging findings, they are unlikely to explain the variation observed across several error awareness studies (Wessel 2012). Instead, disparities in distinguishing ACC activity patterns between aware and unaware errors may be attributed to inadequate statistical power associated with small sample sizes. For example, we have previously found no difference in ACC activity between aware and unaware errors in samples of 13 (Hester et al. 2005) and 16 (Hester, Nestor, et al. 2009) participants; however, we have found greater dACC activity for aware errors in a sample of 27 participants (Hester et al. 2012). Importantly, when the samples of these 3 studies were collated, a significant effect of awareness on dACC activity was observed (Orr and Hester 2012). Insufficient statistical power thus seems to be a robust explanation for these mixed neuroimaging findings (Button et al. 2013; Poldrack et al. 2017). Indeed, low power is a pertinent problem for task-based neuroimaging studies where there are often a small number of observations and few participants (Cremers et al. 2017; Turner et al. 2018). Although recent work has begun to address the reproducibility of brain imaging (Bossier et al. 2020), relatively few functional imaging replication studies have been conducted in this area of research. In light of this shortcoming, the neural correlates of error awareness and the influence of measures of task and individual differences warrant further examination.

Here, we set out to confirm previous investigations using the motor Go/No-Go error awareness task (Hester et al. 2005) in a large, community-based sample. Behavioral performance on the error awareness task and corresponding event-related neuroimaging were used to assess the neural mechanisms associated with error awareness. Based on the reviewed literature, we hypothesize that aware errors will be accompanied by greater activity in a network of regions, including the insula, parietal and midfrontal cortices, and midline structures such as the ACC. Further, we extend upon previous investigations by exploring whether existing findings from clinical cohorts (e.g. ADHD and ASD) are also apparent in larger-scale healthy samples. Specifically, we examined whether the variance in awareness-related neural activity is accounted for by individual differences in dimensional measures of psychopathology, including ADHD, ASD, and impulsivity.

## Materials and methods

### Participants

Participants were recruited via Monash University Clayton campus, social media, and newspaper advertisements along with experimenter networks. All participants were right-handed and had normal or corrected-to-normal vision. Participants were excluded if they were color blind or reported any history of neurological or psychiatric illness, including head injury, previous usage of psychotropic medication, or substance use disorder. All participants provided written informed consent and were reimbursed for participation. The study received approval by the Monash University Human Research Ethics Committee for meeting the research standards prescribed by the National Health and Medical Research Council (CF12/3072—2012001562).

Four hundred and seventy-three participants completed the event-related functional magnetic resonance imaging protocol. Participants were subsequently excluded due to missing functional runs (*n* = 4) or behavioral data (*n* = 22), no signaling of aware errors (*n* = 37), corrupted functional data (*n* = 6), or distorted anatomical data (*n* = 2). The final sample with complete behavioral and neuroimaging data comprised 402 participants (female, 54.22%; *M*_age_ = 23.64 years, standard deviation [SD] = 5.45; age range = 18–50 years). Of those, 20 participants (female, 50%; *M*_age_ = 25.55 years, SD = 7.25) did not have questionnaire data available. Further information on participant age can be found in Supplementary Table 1A.

### Experimental design

Participants were administered a battery of self-report measures designed to assess a comprehensive range of psychopathological characteristics. The battery comprised the Barratt Impulsiveness Scale, Version 11 (BIS-11; Barratt and Patton 1983) to assess impulsivity, Conners’ Adult ADHD Rating Scales—Self Report: Long Version (CAARS—S:L; Conners 1998) to assess ADHD-like behaviors, the Behavioral Inhibition/Activation Systems Scale (BIS/BAS; Carver and White 1994) to measure sensitivity to avoidance and approach motivation, the Autism Spectrum Quotient (AQ; Baron-Cohen et al. 2001) to assess autistic traits, and the Hospital Anxiety and Depression Scale (HADS; Zigmond and Snaith 1983) to assess anxiety and depression traits.

#### Error awareness task

**Fig. 1:**
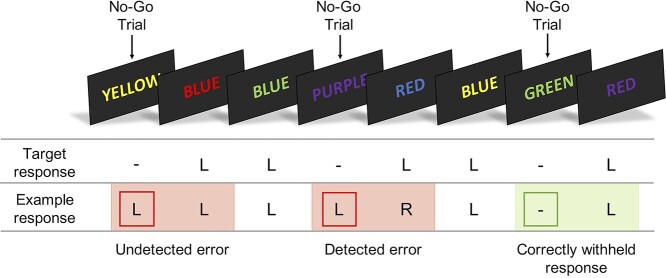
The error awareness task presents a serial stream of color words in a congruent (No-Go trial) or incongruent (Go trial) color. Participants respond to Go trials using a left button press (“L”) while withholding their response to No-Go trials. To indicate error awareness, participants forgo making a standard “Go” response and instead execute a right button press (“R”) on the trial following the commission error. The task comprised 6 blocks, each with 175 trials. Across all blocks, participants were administered 900 Go trials and 150 No-Go trials. All stimuli were presented for 900 ms followed by a 600 ms interstimulus interval.

The error awareness task (see Fig. 1) is a Go/No-Go motor inhibition task that presents a serial stream of color words in a congruent or incongruent color. Previously, we employed an awareness task with 2 competing inhibition rules (a repeat No-Go rule and color No-Go rule). To address concerns that introducing 2 No-Go rules potentially contaminates the blood oxygen level–dependent (BOLD) signal, we opted to remove the repeat No-Go rule. Pilot testing confirmed that unaware error rates with a single No-Go rule were consistent with our previous work (see Supplementary Table 2A for a summary of pilot data). Thus, participants were required to respond to the incongruent trials (Go trials) with a left button press, while withholding their response when the word and color were congruent (No-Go trials). To indicate error awareness, participants were trained to forego making a standard “Go” response and instead execute a right button press following any commission error. Erroneous No-Go trials were those in which a participant failed to withhold a response. To classify erroneous trials for data analysis, unaware errors were those in which the participant responded with a left button press on the No-Go trial and again on the following Go trial. Any deviation from this pattern of response on a No-Go trial or following a No-Go error was classified as an aware error (Fig. 1).

The task comprised 6 blocks, each with 175 trials. Across all blocks, participants were administered 900 Go trials and 150 No-Go trials. All stimuli were presented for 900 ms followed by a 600 ms interstimulus interval. An event-related design was employed, distributing the No-Go trials pseudorandomly throughout the serial presentation of Go trials. Events of interest were adequately separated in order to analyze the correct and failed response inhibition events separately without crosscontamination. The number of Go trials separating No-Go trials ranged between 1 and 12 (*M* = 6.23; SD = 2.55).

### Image acquisition

Scanning was conducted between August 2013 and July 2017 at Monash Biomedical Imaging (Victoria, Australia). Images were acquired using a Siemens Skyra 3-Tesla MRI scanner with a 32-channel head coil. High resolution T1-weighted structural MPRAGE images (time echo [TE] = 2.07 ms, time repetition [TR] = 2,300 ms, FOV = 256 mm, flip angle = 9°, thickness = 1 mm isotropic, sagittal slices) were acquired prior to functional imaging to enable activation localization and for spatial normalization. Functional images were acquired using a gradient-echo pulse sequence (TE = 30 ms, TR = 2,460 ms, FOV = 190 mm, flip angle = 90°, 44 contiguous transversal slices of 3.0 mm thickness). The error awareness task was presented using E-Prime software (version 2.0; Psychology Software Tools) on a Cambridge 32-inch BOLD screen, which was reflected onto a mirror visor positioned in the radio frequency head coil. Participants responded to each stimulus using their right hand, entering their responses using 2 buttons on a 4-button MR-compatible response pad (Fiber-Optic response pads; Current Designs).

### Statistical analysis

#### Behavioral analysis

Behavioral data analyses were undertaken in the programming language R using the “stats” package (R Core Team 2017), with the addition of the “psych” (Revelle 2020), “afex” (Singmann et al. 2020), and “emmeans” (Russell et al. 2020) packages. Effect sizes were calculated using the “effectsize” package (Ben-Shachar et al. 2020). Assumptions were tested, and nonparametric analyses were computed under violations of normality. Greenhouse–Geisser-adjusted degrees of freedom and *P-*values are reported under violations of sphericity. Post hoc tests were undertaken using Tukey’s method for multiple comparisons*. P*-values were otherwise adjusted using Holm procedures. Alpha was set to 0.05 for all analyses. The number of trials available for our behavioral analyses are outlined in Supplementary Table 3A. The full reproducible code for the current results has been made publicly available online (https://osf.io/hrba7/).

The error awareness task is not optimized to analyze response speed adjustments following errors as participants are required to make an awareness button press on the first posterror trial. Switching to the awareness button typically results in abnormally fast reaction times on the Go trials following the error. Response speed adjustments following No-Go trials were therefore determined by calculating the difference in reaction time for the Go trial following the No-Go trial by 3 trials and the Go trial immediately preceding the No-Go trial (a subtraction of the preerror Go reaction time from the third Go reaction time after the No-Go trial).

#### Neuroimaging analysis

Neuroimaging analyses were undertaken using AFNI software (Cox 1996). Data analysis procedures followed those implemented in studies with similar experimental paradigms (e.g. Hester et al. 2012). Behavioral data were used to categorize trial events into the following regressors: correct inhibitions, unaware errors, and aware errors. Activation outside of the brain was removed using edge detection techniques. Following image reconstruction, the time series data were time shifted (using Fourier interpolation) to remove differences in slice acquisition times and were then motion-corrected using 3D volume registration (least-squares alignment of 3 translational and 3 rotational parameters).

Using the BLOCK basis function, separate haemodynamic impulse response functions (IRFs) were computed at 2.46 s temporal resolution for aware errors, unaware errors, and correct inhibitions. To avoid confounding the baseline- and event-related activity estimates, rest and omission errors were included as regressors of no interest. A multiple regression program (3dDeconvolve) determined the best fitting gamma variate function for these IRFs*.* The area under the curve of the gamma variate function was expressed as a percentage of the area under the baseline. The baseline in this design refers to task-related Go-trial processing that remains once the variance of the other events has been removed. The percentage area (event-related activation) map voxels were resampled at 1-mm resolution, then spatially normalized to standard Montreal Neurological Institute (MNI) space, and spatially blurred with a 3-mm isotropic root-mean-squared Gaussian kernel.

Group activation maps were obtained using a paired samples *t-*test (3dttest++) against the null hypothesis of no event-related activation differences between aware and unaware errors. Significant voxels passed a voxel-wise statistical threshold (*t* = 6.60, *P* = 1.0 × 10^−10^) and were required to be part of a 250-μL cluster of significant contiguous voxels. This method of combining probability and cluster thresholding sought to maximize power while minimizing the likelihood of false-positives. ANFI’s 3dClustSim was provided with the number of voxels in the group map, the spatial correlation of the voxels, and the voxel-wise threshold. A series of Monte Carlo simulations (10,000 iterations) were then undertaken to determine the frequency of varying sized clusters produced by chance. From this frequency distribution, we selected the cluster size that occurred <1% of the time by chance, to provide a threshold of *P* = 0.010, corrected. Using this method for the current sample resulted in a highly liberal cluster-wise threshold (<1 μL). We thus opted for a cluster-wise threshold of 250 μL as it is far more conservative and is moreover comparable with previous studies (e.g. Hester et al. 2005). Mean activity estimates for each event were derived for clusters in the whole-brain map using the program 3DRoiStats. The estimates were used in assessing the relationship between neural activity and measures of task performance and individual differences.

#### Linking neural activity to psychopathological traits

Lasso (least absolute shrinkage and selection operator) regression was employed to determine a subset of the dimensional psychopathological measures that best predict error awareness and mean activity estimates for the insula cortex, ACC, supramarginal gyrus (SMG), and middle frontal gyrus. Lasso is a modified form of least-squares regression that applies a regularization parameter (λ) to determine the variables that best predict the outcome measure (Tibshirani 1996). The regularization parameter shrinks coefficients to 0 for irrelevant covariates in order to minimize prediction error and reduce overfitting. The optimal penalty term was determined using a 10-fold crossvalidation. By enforcing sparsity, lasso regression provides a principled way of identifying a subset of predictors that have the strongest influence on the dependent variable (Tibshirani 1996). Lasso generalized linear models were computed in the programming language R (R Core Team 2017) using the “glmnet” package (Friedman et al. 2010). The main independent variables were subscale scores from each of the aforementioned psychopathological questionnaires. Although a whole-brain approach was used to explore the regions associated with awareness, a more focused subset of areas was selected as dependent variables to investigate how awareness activity is related to psychopathological traits. Dependent variables were therefore error awareness and mean aware activity estimates for 4 clusters identified in our imaging analysis (insula cortex, ACC, SMG, and middle frontal gyrus). These clusters were selected due to theoretical relevance and previous findings of sensitivity to error awareness (Harsay et al. 2012; Orr and Hester 2012; Klein et al. 2013). Five separate models were computed, 1 for each dependent variable. The analysis does not allow missing data, therefore, cases with missing values were omitted. Little’s test indicated that data were missing completely at random, χ^2^(155) = 173.53, *P* = 0.155. All variables were standardized prior to analysis to generate *Z*-scores. A test statistic or *P-*value for lasso regression is still under development (Lockhart et al. 2014). Further, given the interest here is predictive performance and not statistical inference, results are presented as standardized regression weights alone. To determine the robustness of the variable selection, each lasso model was computed on 500 bootstrap samples. The percentage of nonzero bootstrap samples is reported for each variable alongside the coefficients.

## Results

### Behavioral results

Performance indices are summarized in Table 1. Participants correctly withheld 53.59% of their responses on No-Go trials and were aware of 86.47% of commission errors (error awareness range = 21.74–99.11%). There was a nonsignificant weak association between the awareness of errors and overall inhibition performance, *r_s_* = −0.10, *P =* 0.055. A repeated measures ANOVA revealed that the speed of response was significantly related to trial type, *F*(2, 798) = 21.37, *P* < 0.001, η*_p_*^2^ = 0.05. Post hoc tests using the Tukey method for multiple comparisons indicated that reaction times were significantly faster for aware errors than for either unaware errors, *t*(798) = −4.22, *P* < 0.001, *d* = −.23, 95% confidence interval (CI) (−0.30, −0.16), or correct Go responses, *t*(798) = −6.44, *P* < 0.001, *d* = −.15, 95% CI (−0.22, −0.08). There was no significant difference in the reaction time between correct responses and unaware errors, *t*(798) = 2.22, *P =* 0.069, *d* = −.08, 95% CI (−0.15, −0.01).

**Table 1:** Behavioral performance: inhibition accuracy, error awareness, and reaction time on the error awareness task.

Category	Mean (SD)
Inhibition accuracy %	53.59 (19.31)
Total errors	66.45 (29.05)
Error awareness %	86.47 (11.74)
Reaction time (ms)	
Go trial	517.66 (80.60)
Aware trial	489.14 (107.28)
Unaware trial	507.37 (117.33)
Post-No-Go reaction time adjustment (ms)^a^	
Aware error	+5.94 (49.66)
Unaware error	+19.22 (93.21)
Correct inhibition	+7.89 (44.24)

a Post-No-Go reaction time—pre-No-Go reaction time. Post-No-Go reaction time is taken from the Go trial that succeeds the No-Go error by 3 trials.

A repeated measures ANOVA was computed to compare reaction time adjustments across No-Go responses (correct inhibition, unaware error, and aware error). The results revealed an effect of No-Go response type on post-No-Go reaction time, *F*(1.52, 598.43) = 4.85, *P* = 0.015, η*_p_*^2^ = 0.01. Post hoc tests indicated greater slowing of responses following unaware errors (+19 ms) compared to aware errors (+5 ms), *t*(786) = 2.91, *P* = 0.010, *d* = −.48, 95% CI (−0.56, −0.41), and correct No-Go responses (+7 ms), *t*(786) = 2.41, *P =* 0.043, *d* = −.40, 95% CI (−0.47, −0.33). There was no significant difference in post-No-Go reaction adjustments between correct responses and aware errors, *t*(786) = 0.50, *P* = 0.869, *d* = 0.08, 95% CI (0.01, 0.15).

### Neuroimaging results

**Fig. 2:**
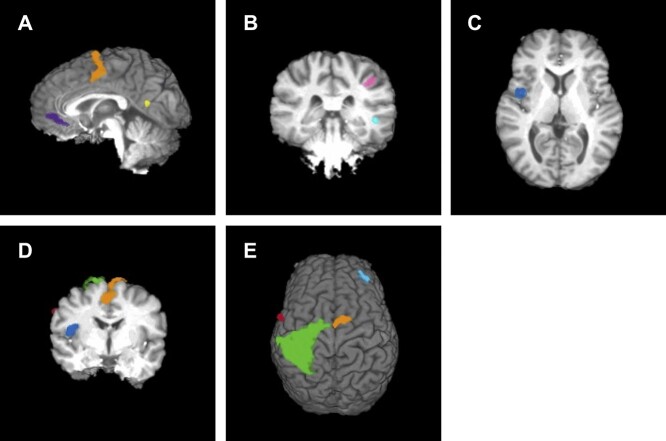
Clusters associated with greater BOLD signal for aware errors than unaware errors*.* A) Sagittal slice at *x* = 0. Purple cluster centered on left ACC, orange cluster centered on left SMA, yellow cluster centered on left precuneus; B) coronal slice at *y* = 30. Blue cluster centered on right superior temporal gyrus, pink cluster centered on right SMG; C) axial slice at *z* = 10. Blue cluster centered on left insula; D) coronal slice at *y* = 5. Blue cluster centered on left insula, red cluster centered on left precentral gyrus, orange cluster centered on left SMA, green cluster centered on left postcentral gyrus; E) green cluster centered on right postcentral gyrus, orange cluster centered on left SMA, red cluster centered on left precentral gyrus, blue cluster centered on right middle frontal gyrus.

The event-related functional analysis revealed 17 clusters that differentiated aware errors from unaware errors (Table 2). Aware errors were accompanied by greater activity in the left insula cortex (Fig. 2C and D), the SMG (Fig. 2B), and midline structures such as the left supplementary motor area (SMA), left ACC, and bilateral precuneus (Fig. 2A). It should be noted that while the center of mass of activity in the SMA and ACC falls within the left hemisphere, the lateral extent of these clusters was bilateral.

**Table 2:** Regions that showed significantly greater BOLD signal for aware errors than unaware errors.

Structure	Vol. μL	HS	Center of mass (MNI coordinates)		
			*x*	*y*	*z*
Postcentral gyrus	30,420	L	−38	−32	54
Cerebellum	7,778	R	23	−50	−27
Cerebellum	4,055	L	−30	−52	−31
SMA^a^	2,849	L	1	−6	58
Cerebellum	2,401	R	21	−49	−57
ACC^a^	1,784	L	−2	41	−3
Insula	1,521	L	−40	−2	13
SMG	1,368	R	39	−37	43
Cerebellum	1,085	L	−34	−44	−59
Middle frontal gyrus	703	L	−32	37	35
Middle frontal gyrus	687	R	32	44	38
SMG	585	R	56	−43	26
Precuneus	554	R	7	−73	42
Precuneus	541	L	−5	−57	13
Superior temporal gyrus	394	R	46	−29	−3
Precentral gyrus	375	L	−57	2	33
Precuneus	252	L	−11	−70	47

Note. Positive values for *x*, *y*, and *z* coordinates denote locations that are right, posterior, and superior relative to the anterior commissure, respectively.

a While the center of mass of activity in the SMA and ACC falls within the left hemisphere, the lateral extent of these clusters was bilateral.

ACC activity was not robustly correlated with behavioral measures that are typically related to error awareness. That is, the speed of error commission was not significantly associated with the degree of ACC activity for either aware, *r_s_* = −0.09, *P* = 0.061, or unaware errors, *r_s_* = −0.06, *P* = 0.194. Further, we did not find evidence in support of an association between inhibition performance and ACC activity related to aware errors, *r_s_* = 0.01, *P* = 0.890, or error awareness rate, *r_s_* = 0.09, *P* = 0.063. Likewise, we found no evidence for an association between posterror reaction time adjustments and ACC activity for aware errors, *r_s_* = −0.06, *P* = 0.233, or unaware errors, *r_s_* = −0.08, *P* = 0.096. The difference between BOLD activity in the ACC associated with aware errors and that associated with unaware errors was also not found to be significantly related to the speed of aware errors, *r_s_* = −0.02, *P* = 1.00, and unaware errors, *r_s_* = 0.01, *P* = 1.00, or posterror adjustments in reaction time following aware errors, *r_s_* = −0.03, *P* = 1.00, and unaware errors, *r_s_* = 0.09, *P* = 0.284.

The association between neural activity and performance indices was further assessed. Inhibition performance was found to correlate positively with the BOLD activity associated with aware errors in the insula, *r_s_* = 0.12, *P* = 0.030, and right SMG, *r_s_* = 0.25, *P* < 0.001. Only the left middle frontal gyrus, *r_s_* = 0.17, *P* = 0.010, and the SMA, *r_s_* = 0.15, *P* = 0.030, were found to correlate significantly with aware error reaction time. Postaware reaction time adjustments were associated with activity in the SMA, *r_s_* = 0.15, *P* = 0.030, right SMG, *r_s_* = 0.19, *P* < 0.001, right superior temporal gyrus, *r_s_* = 0.14, *P* = 0.040, and left and right middle frontal gyri, *r_s_* = 0.17, *P* = 0.010, and *r_s_* = 0.21, *P* < 0.001, respectively, such that greater activity in these regions correlated with slower reaction time on the posterror trial. For unaware errors, neither the speed of the erroneous response nor posterror reaction time adjustments were found to significantly correlate with BOLD activity in any of the 17 clusters.

### Lasso regression

Lasso regression results for each model are summarized in Table 3. Error awareness was found to be predicted by impulsivity, namely motor and planning scores from the BIS-11 (Barratt and Patton 1983), and behavioral inhibition score from the BIS/BAS (Carver and White 1994). The most important predictors of insula activity were attention to detail and imagination scores from the AQ (Baron-Cohen et al. 2001) and HADS depression score (Zigmond and Snaith 1983). No variable was found to predict ACC activity, while all variables except attention switching from the AQ, and attention, self-concept, and DSM attention from the CAARS (Conners 1998) were found to predict SMG activity.

**Table 3:** Lasso regression coefficients.

	Outcome									
	Error awareness		L-insula		ACC		R-SMG		R-middle frontal gyrus	
Predictor	Lasso coeff.	Nonzero (%)	Lasso coeff.	Nonzero (%)	Lasso coeff.	Nonzero (%)	Lasso coeff.	Nonzero (%)	Lasso coeff.	Nonzero (%)
BIS-11—attentional	–	56	–	56	–	26	−0.05	58	–	33
BIS-11—motor	−0.07	86	–	46	–	30	0.11	78	0.05	74
BIS-11—nonplanning	−0.03	90	–	38	–	48	−0.01	52	–	34
AQ—social skill	–	48	–	40	–	56	−0.15	82	–	46
AQ—attention switching	–	68	–	50	–	22	–	54	–	37
AQ—attention to detail	–	64	.0003	60	–	44	0.04	64	–	47
AQ—communication	–	58	–	56	–	20	0.16	92	0.05	71
AQ—imagination	–	54	0.02	76	–	34	0.03	64	0.13	92
BIS/BAS—BAS drive	–	68	–	56	–	30	0.04	66	0.05	72
BIS/BAS—BAS fun	–	50	–	42	–	24	−0.12	74	–	25
BIS/BAS—BAS reward	–	62	–	42	–	34	0.01	54	–	41
BIS/BIS—BIS score	0.04	90	–	68	–	30	0.03	60	0.01	53
HADS—anxiety	–	52	–	50	–	30	0.02	64	–	49
HADS—depression	–	52	0.10	90	–	34	0.19	98	–	79
CAARS—attention	–	42	–	38	–	22	–	38	–	43
CAARS—hyperactivity	–	46	–	52	–	24	0.11	54	–	25
CAARS—impulsivity	–	48	–	48	–	22	−0.04	60	–	37
CAARS—self-concept	–	46	–	42	–	54	–	36	–	32
CAARS—DSM attention	–	34	–	50	–	20	–	42	−0.05	49
CAARS—DSM hyperactivity	–	38	–	40	–	22	−0.14	60	–	23
CAARS—DSM ADHD	–	6	–	4	–	6	−0.01	20	–	24
CAARS—index	–	64	–	18	–	12	−0.02	40	–	24

Note. Results are presented as standardized regression coefficients. To determine the robustness of the variable selection, each lasso model was computed on 500 bootstrap samples. The percentage of nonzero bootstrap samples is reported for each variable alongside the coefficients.

Regarding the questionnaire measures, it is worth noting that only a very small fraction of participants reported clinically relevant scores (see Supplementary Table 4A for descriptive statistics). The largest psychopathological subsample were individuals scoring in the clinical range for HADS anxiety (*n* = 196). Therefore, we compared the subsample of individuals meeting the cutoff for clinical levels of anxiety with those who did not. Corroborating the results of the lasso regressions, no difference was found between the groups in mean insula, *t*(379) = 0.78, *P* > 0.990, ACC, *t*(379) = 0.14, *P* > 0.990, SMG, *t*(379) = 2.10, *P* = 0.147, and middle frontal gyrus activity, *t*(379) = 0.77, *P* < 0.990.

## Discussion

The current study aimed to establish the robustness of previous findings on the neural correlates of error awareness. Here, we have discerned greater aware-related activity in a network of regions, including the insula cortex, ACC, SMA, and SMG. Further, individual differences in error-related neural activity were found to be related to indices of task performance in a select few regions, including the insula, SMA and SMG. Moreover, we found that certain measures of psychopathology—namely impulsiveness and depression—explained variance in aware-related activity in a subset of these regions.

Although the ACC has been implicated in several studies on performance monitoring, differentiation of activity in this region with error awareness has been largely unreported (but, see Hester et al. 2012). Our study has shown greater ACC activation—across the dACC and rACC—for aware errors than unaware errors, suggesting a sensitivity of the ACC to awareness. This supports claims that insufficient statistical power may underlie the discrepancy in previous findings (Wessel 2012). Although the precise role of the ACC in error processing is unknown, there is a general consensus that the ACC—particularly the dACC—monitors ongoing behavior and navigates the selection and evaluation of goal-directed behaviors (Holroyd and Yeung 2012). In particular, it is purported to respond to outcomes that are worse than expected and may signal the need for an adjustment in the strategy to reach the desired goal (Holroyd and Coles 2002; Bryden et al. 2011). Our finding of greater rACC activation is not typically reported in error awareness studies; however, the rACC has been proposed to be a neuronal generator of the Pe—an ERP associated with error awareness (Herrmann et al. 2004; Van Boxtel et al. 2005). While the rACC may be differentially involved in posterror processing, as evidenced by the Pe, further work is ultimately needed to discern precisely how the rACC contributes to awareness. Taken together, it is plausible that the ACC presents a threshold-like relationship to awareness and posterror processes, whereby a certain level of activity is sufficient to elicit error detection and posterror adaptation, but the overall level of activity is not tightly coupled to these processes (Orr and Hester 2012). This is moreover consistent with the absence of a relationship between the individual differences in aware-related ACC activity and behavioral adjustments in our study. Thus, ACC activity may covary with error commission and contribute to error awareness such that it is facilitating goal attainment, however, may not be solely responsible for eliciting awareness and posterror alterations.

Contrastingly, the insula cortex appears to be consistently modulated by error awareness. The insula has been proposed to be engaged in a number of processes, however, its role in interoceptive awareness has taken prominence in recent decades. In particular, the insula integrates autonomic information with salient events such as errors (Klein et al. 2013). Insula activity during aware errors may therefore be explained by interoceptive awareness of greater autonomic responses to aware errors (Craig 2009). Interestingly, concurrent insula and ACC activity during performance monitoring is a robust finding (Craig 2009; Ham et al. 2013). Although these structures are distinct, they have been purported to form a salience network which has been associated with interoceptive autonomic domains and the control of goal-directed behaviors (Dosenbach et al. 2007). Indeed, previous studies have found the ACC to be associated with autonomic engagement during aware error processing (Harsay et al. 2018). The relationship between the ACC and insula may explain how the ACC potentially mediates error awareness. Specifically, insula activity may represent awareness, while ACC activity represents the control of directed effort. That is, error-related activity in the ACC may feedforward into the insula which may be more directly responsible for error awareness.

Consistent with previous findings, aware errors were associated with greater activity in the right SMG. The SMG is purported to be connected to the ACC and middle frontal gyrus via the dorsal branch of the superior longitudinal fasciculus (SLF1; Ramos-Fresnedo et al. 2019). Recent research has demonstrated that individual differences in the SLF1 underpin an individual’s evidence accumulation capacity (Brosnan et al. 2020). This is pertinent given that current views on error awareness operate in line with an evidence accumulation account (Ullsperger et al. 2010). The emergence of error awareness is said to coincide with the accumulation of evidence above a response criterion threshold (Murphy et al. 2012). Given the SMG, ACC, and middle frontal gyrus were found to contribute to error awareness in the current study, it is plausible that connectivity between these regions might be a critical determinant of an individual’s error awareness.

It is also worth considering that the inferior parietal lobe—which in part comprises the SMG—has been proposed to form a network with the ACC and insula and together are associated with the salience of an event (Harsay et al. 2012). The parietal lobe, in particular, is suggested to act on salient events and likely works to direct and maintain the location of attention (Corbetta and Shulman 2002). Errors are arguably salient as they are infrequent and useful in that they redirect a participant’s attention to current task goals. Indeed, consistent with this orienting account, we found elevated SMG activity to be correlated with slower reaction times following aware errors. This finding aligns with previous work which has found that correct trials following an error show heightened activation of the inferior parietal lobe, coinciding with increased posterror slowing (Marco-Pallarés et al. 2008).

Although error awareness rate did not appear to be associated with inhibition performance, we found a relationship between inhibition and aware-related activity in the insula and SMG. This is interesting given that error-related activity in the insula and inferior parietal lobe have previously been found to predict successful inhibition on the following No-Go trial (Hester, Madeley, et al. 2009), suggesting a shared neural system between error awareness and successful response inhibition. Previously, we speculated that inclusion of 2 inhibition contingencies might disrupt the relationship between error awareness and future performance, reflecting the role of the ACC as a reinforcement learning signal (Orr and Hester 2012). We therefore opted to include only 1 inhibition rule in the current design. Despite this task change, we found no relationship between error awareness and inhibition performance. It is thus plausible that error awareness facilitates performance only under context-specific conditions—where there is a more direct contingency between an error and future performance. Since there was no direct contingency here, with performance not influencing the sequence of trials that followed, it is likely any increases in conservatism of responding are loosely, if at all, reflective of sustained changes in performance strategy.

While we examined posterror reaction time adjustments, it is worth considering that the error awareness task is not optimized for this analysis. Specifically, participants are required to make an awareness button press on the first posterror trial. To minimize this confound, we excluded the first 2 posterror trials from our posterror slowing analysis, however, we still found greater slowing following unaware errors. While studies on posterror slowing and error awareness have generated mixed evidence (van Gaal et al. 2009; Hewig et al. 2011; Endrass et al. 2012; Hoonakker et al. 2016), the finding of greater slowing following unaware errors appears to be exclusive to studies employing the error awareness task. Given that posterror reaction time did not return to baseline by the third posterror trial, it seems plausible that unaware errors are accompanied by the continued anticipation of an impending No-Go trial, resulting in slowed responses. Our finding of greater slowing following unaware errors is therefore likely to be a task-specific phenomenon rather than a reflection of deliberate posterror behavioral adjustments. To reconcile these findings, we require a task that obviates the need for an error awareness button press on the posterror trial and offers more events (i.e., aware and unaware errors) per individual.

To examine the influence of dimensional measures of psychopathology on error awareness and related neural activity in 4 selected clusters (insula, ACC, SMG, and middle frontal gyrus), we ran a series of lasso regressions. The most robust positive predictors of error awareness were impulsivity-related measures, specifically motor and nonplanning impulsiveness. This is consistent with the finding that disorders marked by deficits in impulsiveness, such as ADHD and substance use disorder, have been shown to have impaired error awareness (O’Connell et al. 2009; Charles et al. 2017). Error awareness was also found to be positively predicted by behavioral inhibition system score which reflects the motivation to avoid adverse outcomes and is purported to be predictive of the affective and behavioral responses after incentives and threats (Johnson et al. 2003). Moreover, the relationships between aware-related insula and SMG activity were most notably found to be positively predicted by depressive symptoms. Although some studies have found a heightened Pe—an ERP which is suggested to index error awareness—to be related to depressive symptoms (Mies et al. 2011; Mueller et al. 2015), others have found no such relationship (Compton et al. 2008). It has been reported, however, that depressed individuals display greater activity in the insula in response to negative stimuli than healthy controls (Hamilton et al. 2012). The heightened sensitivity to failure and negative information which is proposed to underlie clinical levels of depression may in part explain why aware-related activity in these regions is related to depressive traits in a nonclinical sample.

## Conclusion

Our event-related analysis of a large sample revealed a network of regions, including the insula cortex, SMG, and midline structures such as the ACC and SMA that show greater BOLD signal change for aware errors compared to unaware errors. The most parsimonious account of error awareness is that it is likely the result of the accumulative efforts of these systems which may not all individually drive awareness.

## Supplementary Material

supplementary_material_bhac077

## Data Availability

Data are available upon reasonable request and all scripts required for the current results have been made publicly available online at the Open Science Framework (https://osf.io/hrba7/).

## References

[ref1] Baron-Cohen S, Wheelwright S, Skinner R, Martin J, Clubley E. The autism-spectrum quotient (AQ): evidence from Asperger syndrome/high-functioning autism, males and females, scientists and mathematicians. J Autism Dev Disord. 2001:31:5–17. 11439754 10.1023/a:1005653411471

[ref2] Barratt ES, Patton JH. Impulsivity: cognitive, behavioral, and psychophysiological correlates. In: Zuckerman M, editors. editor Biological bases of sensation seeking, impulsivity and anxiety. Hillsdale (NJ): Erlbaum; 1983. pp. 77–116

[ref3] Ben-Shachar M, Lüdecke D, Makowski D. effectsize: estimation of effect size indices and standardized parameters. J Open Source Softw. 2020:5:2815.

[ref4] Bossier H, Roels SP, Seurinck R, Banaschewski T, Barker GJ, Bokde ALW, Quinlan EB, Desrivières S, Flor H, Grigis A, et al. The empirical replicability of task-based fMRI as a function of sample size. NeuroImage. 2020:212:116601. 32036019 10.1016/j.neuroimage.2020.116601

[ref5] Brosnan MB, Sabaroedin K, Silk T, Genc S, Newman DP, Loughnane GM, Fornito A, O'Connell RG, Bellgrove MA. Evidence accumulation during perceptual decisions in humans varies as a function of dorsal frontoparietal organization. Nat Hum Behav. 2020:4:844–855. 32313233 10.1038/s41562-020-0863-4

[ref6] Bryden DW, Johnson EE, Tobia SC, Kashtelyan V, Roesch MR. Attention for learning signals in anterior cingulate cortex. J Neurosci. 2011:31:18266. 22171031 10.1523/JNEUROSCI.4715-11.2011 PMC3285822

[ref7] Button KS, Ioannidis JPA, Mokrysz C, Nosek BA, Flint J, Robinson ESJ, Munafò MR. Power failure: why small sample size undermines the reliability of neuroscience. Nat Rev Neurosci. 2013:14:365–376. 23571845 10.1038/nrn3475

[ref8] Carver CS, White TL. Behavioral inhibition, behavioral activation, and affective responses to impending reward and punishment: the BIS/BAS scales. J Pers Soc Psychol. 1994:67:319–333.

[ref9] Charles L, Gaillard R, Amado I, Krebs M-O, Bendjemaa N, Dehaene S. Conscious and unconscious performance monitoring: evidence from patients with schizophrenia. NeuroImage. 2017:144:153–163. 27670235 10.1016/j.neuroimage.2016.09.056

[ref10] Compton RJ, Lin M, Vargas G, Carp J, Fineman SL, Quandt LC. Error detection and posterror behavior in depressed undergraduates. Emotion. 2008:8:58–67. 18266516 10.1037/1528-3542.8.1.58

[ref11] Conners CK . Rating scales in attention-deficit/hyperactivity disorder: use in assessment and treatment monitoring. J Clin Psychiatry. 1998:59:24–30. 9680050

[ref12] Corbetta M, Shulman GL. Control of goal-directed and stimulus-driven attention in the brain. Nat Rev Neurosci. 2002:3:201–215. 11994752 10.1038/nrn755

[ref13] Cox RW . AFNI: software for analysis and visualization of functional magnetic resonance neuroimages. Comput Biomed Res. 1996:29:162–173. 8812068 10.1006/cbmr.1996.0014

[ref14] Craig AD . How do you feel — now? The anterior insula and human awareness. Nat Rev Neurosci. 2009:10:59–70. 19096369 10.1038/nrn2555

[ref15] Cremers HR, Wager TD, Yarkoni T. The relation between statistical power and inference in fMRI. PLoS One. 2017:12:e0184923. 29155843 10.1371/journal.pone.0184923 PMC5695788

[ref16] Debener S, Ullsperger M, Siegel M, Fiehler K, von Cramon DY, Engel AK. Trial-by-trial coupling of concurrent electroencephalogram and functional magnetic resonance imaging identifies the dynamics of performance monitoring. J Neurosci. 2005:25:11730–11737. 16354931 10.1523/JNEUROSCI.3286-05.2005 PMC6726024

[ref17] Dhar M, Wiersema JR, Pourtois G. Cascade of neural events leading from error commission to subsequent awareness revealed using EEG source imaging. PLoS One. 2011:6:1–12. 10.1371/journal.pone.0019578 PMC3088685 21573173

[ref18] Dosenbach NUF, Fair DA, Miezin FM, Cohen AL, Wenger KK, Dosenbach RAT, Fox MD, Snyder AZ, Vincent JL, Raichle ME, et al. Distinct brain networks for adaptive and stable task control in humans. Proc Natl Acad Sci U S A. 2007:104:11073–11078. 17576922 10.1073/pnas.0704320104 PMC1904171

[ref19] Endrass T, Reuter B, Kathmann N. ERP correlates of conscious error recognition: aware and unaware errors in an antisaccade task. Eur J Neurosci. 2007:26:1714–1720. 17880402 10.1111/j.1460-9568.2007.05785.x

[ref20] Endrass T, Klawohn J, Preuss J, Kathmann N. Temporospatial dissociation of Pe subcomponents for perceived and unperceived errors. Front Hum Neurosci. 2012:6:1–10. 22737113 10.3389/fnhum.2012.00178 PMC3381446

[ref21] Falkenstein M, Hohnsbein J, Hoormann J, Blanke L. Effects of crossmodal divided attention on late ERP components. II. Error processing in choice reaction tasks. Electroencephalogr Clin Neurophysiol. 1991:78:447–455. 1712280 10.1016/0013-4694(91)90062-9

[ref22] Friedman J, Hastie T, Tibshirani R. Regularization paths for generalized linear models via coordinate descent. J Stat Softw. 2010:33:1–22. 20808728 PMC2929880

[ref24] Gehring WJ, Goss B, Coles MGH, Meyer DE, Donchin E. A neural system for error detection and compensation. Psychol Sci. 1993:4:385–390.

[ref25] Ham T, Leff A, de Boissezon X, Joffe A, Sharp DJ. Cognitive control and the salience network: an investigation of error processing and effective connectivity. J Neurosci. 2013:33:7091–7098. 23595766 10.1523/JNEUROSCI.4692-12.2013 PMC6618896

[ref26] Hamilton JP, Etkin A, Furman DJ, Lemus MG, Johnson RF, Gotlib IH. Functional neuroimaging of major depressive disorder: a meta-analysis and new integration of baseline activation and neural response data. Am J Psychiatry. 2012:169:693–703. 22535198 10.1176/appi.ajp.2012.11071105 PMC11889638

[ref27] Harsay HA, Spaan M, Wijnen JG, Ridderinkhof KR. Error awareness and salience processing in the oddball task: shared neural mechanisms. Front Hum Neurosci. 2012:6:1–20. 22969714 10.3389/fnhum.2012.00246 PMC3427876

[ref28] Harsay HA, Cohen MX, Spaan M, Weeda WD, Nieuwenhuis S, Ridderinkhof KR. Error blindness and motivational significance: shifts in networks centering on anterior insula co-vary with error awareness and pupil dilation. Behav Brain Res. 2018:355:24–35. 29107022 10.1016/j.bbr.2017.10.030

[ref29] Herrmann MJ, Römmler J, Ehlis A-C, Heidrich A, Fallgatter AJ. Source localization (LORETA) of the error-related-negativity (ERN/Ne) and positivity (Pe). Brain Res Cogn. 2004:20:294–299. 10.1016/j.cogbrainres.2004.02.013 15183400

[ref30] Hester R, Foxe JJ, Molholm S, Shpaner M, Garavan H. Neural mechanisms involved in error processing: a comparison of errors made with and without awareness. NeuroImage. 2005:27:602–608. 16024258 10.1016/j.neuroimage.2005.04.035

[ref31] Hester R, Nestor L, Garavan H. Impaired error awareness and anterior cingulate cortex hypoactivity in chronic cannabis users. Neuropsychopharmacology. 2009:34:2450–2458. 19553917 10.1038/npp.2009.67 PMC2743772

[ref32] Hester R, Madeley J, Murphy K, Mattingley JB. Learning from errors: error-related neural activity predicts improvements in future inhibitory control performance. J Neurosci. 2009:29:7158–7165. 19494138 10.1523/JNEUROSCI.4337-08.2009 PMC6666478

[ref33] Hester R, Nandam LS, O’Connell RG, Wagner J, Strudwick M, Nathan PJ, Mattingley JB, Bellgrove MA. Neurochemical enhancement of conscious error awareness. J Neurosci. 2012:32:2619–2627. 22357846 10.1523/JNEUROSCI.4052-11.2012 PMC6621889

[ref34] Hewig J, Coles MGH, Trippe RH, Hecht H, Miltner WHR. Dissociation of Pe and ERN/Ne in the conscious recognition of an error. Psychophysiology. 2011:48:1390–1396. 21534985 10.1111/j.1469-8986.2011.01209.x

[ref35] Hoffmann S, Beste C. A perspective on neural and cognitive mechanisms of error commission. Front Behav Neurosci. 2015:9:1–16. 25784865 10.3389/fnbeh.2015.00050 PMC4347623

[ref36] Holroyd CB, Coles MG. The neural basis of human error processing: reinforcement learning, dopamine, and the error-related negativity. Psychol Rev. 2002:109:679–709. 12374324 10.1037/0033-295X.109.4.679

[ref37] Holroyd CB, Yeung N. Motivation of extended behaviors by anterior cingulate cortex. Trends Cogn Sci. 2012:16:122–128. 22226543 10.1016/j.tics.2011.12.008

[ref38] Hoonakker M, Doignon-Camus N, Bonnefond A. Performance monitoring mechanisms activated before and after a response: a comparison of aware and unaware errors. Biol Psychol. 2016:120:53–60. 27568326 10.1016/j.biopsycho.2016.08.009

[ref39] Johnson SL, Turner RJ, Iwata N. BIS/BAS levels and psychiatric disorder: an epidemiological study. J Psychopathol Behav Assess. 2003:25:25–36.

[ref40] Klein TA, Endrass T, Kathmann N, Neumann J, von Cramon DY, Ullsperger M. Neural correlates of error awareness. NeuroImage. 2007:34:1774–1781. 17185003 10.1016/j.neuroimage.2006.11.014

[ref41] Klein TA, Ullsperger M, Danielmeier C. Error awareness and the insula: links to neurological and psychiatric diseases. Front Hum Neurosci. 2013:7:14. 23382714 10.3389/fnhum.2013.00014 PMC3563042

[ref42] Lockhart R, Taylor J, Tibshirani RJ, Tibshirani R. A significance test for the LASSO. Ann Stat. 2014:42:413–468. 25574062 10.1214/13-AOS1175 PMC4285373

[ref43] Maier M, Steinhauser M, Hübner R. Is the error-related negativity amplitude related to error detectability? Evidence from effects of different error types. J Cogn Neurosci. 2008:20:2263–2273. 18457501 10.1162/jocn.2008.20159

[ref44] Marco-Pallarés J, Camara E, Münte TF, Rodríguez-Fornells A. Neural mechanisms underlying adaptive actions after slips. J Cogn Neurosci. 2008:20:1595–1610. 18345985 10.1162/jocn.2008.20117

[ref45] Masina F, Tarantino V, Vallesi A, Mapelli D. Repetitive TMS over the left dorsolateral prefrontal cortex modulates the error positivity: an ERP study. Neuropsychologia. 2019:133:107153. 31398426 10.1016/j.neuropsychologia.2019.107153

[ref46] Mies GW, van der Veen FM, Tulen JHM, Birkenhäger TK, Hengeveld MW, van der Molen MW. Drug-free patients with major depression show an increased electrophysiological response to valid and invalid feedback. Psychol Med. 2011:41:2515–2525. 21733223 10.1017/S0033291711000778

[ref47] Mueller EM, Pechtel P, Cohen AL, Douglas SR, Pizzagalli DA. Potentiated processing of negative feedback in depression is attenuated by adhedonia. Depress Anxiety. 2015:32:296–305. 25620272 10.1002/da.22338 PMC4374007

[ref48] Murphy P, Robertson I, Allen D, Hester R, O'Connell R. An electrophysiological signal that precisely tracks the emergence of error awareness. Front Hum Neurosci. 2012:6:65. 22470332 10.3389/fnhum.2012.00065 PMC3314233

[ref49] Nieuwenhuis S, Ridderinkhof KR, Blom J, Band GPH, Kok A. Error-related brain potentials are differentially related to awareness of response errors: evidence from an antisaccade task. Psychophysiology. 2001:38:752–760. 11577898

[ref50] O’Connell RG, Bellgrove MA, Dockree PM, Lau A, Hester R, Garavan H, Fitzgerald M, Foxe JJ, Robertson IH. The neural correlates of deficient error awareness in attention-deficit hyperactivity disorder (ADHD). Neuropsychologia. 2009:47:1149–1159. 19350709 10.1016/j.neuropsychologia.2009.01.011

[ref51] O'Connell RG, Dockree PM, Bellgrove MA, Kelly SP, Hester R, Garavan H, Robertson IH, Foxe JJ. The role of cingulate cortex in the detection of errors with and without awareness: a high-density electrical mapping study. Eur J Neurosci. 2007:25:2571–2579. 17445253 10.1111/j.1460-9568.2007.05477.x

[ref52] Orr C, Hester R. Error-related anterior cingulate cortex activity and the prediction of conscious error awareness. Front Hum Neurosci. 2012:6:177. 22723775 10.3389/fnhum.2012.00177 PMC3377932

[ref53] Poldrack RA, Baker CI, Durnez J, Gorgolewski KJ, Matthews PM, Munafò MR, Nichols TE, Poline J-B, Vul E, Yarkoni T. Scanning the horizon: towards transparent and reproducible neuroimaging research. Nat Rev Neurosci. 2017:18:115–126. 28053326 10.1038/nrn.2016.167 PMC6910649

[ref54] R Core Team . R: a language and environment for statistical computing. Vienna, Austria: R Foundation for Statistical Computing; 2017.

[ref55] Ramos-Fresnedo A, Segura-Duran I, Chaichana KL, Pillai JJ. Chapter 2—supratentorial White matter tracts. In: Chaichana K, Quiñones-Hinojosa A, editors. editorsComprehensive overview of modern surgical approaches to intrinsic brain tumors. Academic Press; 2019. pp. 23–35.

[ref56] Revelle W. Psych: procedures for personality and psychological research. R package version 2.0.12. Evanston, Illinois: Northwestern University; 2020.

[ref57] Russell L, Henrik S, Love J, Buerkner P, Herve M. Package ‘emmeans’: estimated marginal means, aka least-squares means. R package version 1.4.8. 2020.

[ref58] Scheffers MK, Coles MG. Performance monitoring in a confusing world: error-related brain activity, judgments of response accuracy, and types of errors. J Exp Psychol Hum Percept Perform. 2000:26:141–151. 10696610 10.1037//0096-1523.26.1.141

[ref59] Shalgi S, Deouell LY. Is any awareness necessary for an Ne? Front Hum Neurosci. 2012:6:1–15. 22593739 10.3389/fnhum.2012.00124 PMC3343320

[ref60] Singmann H, Bolker B, Westfall J, Aust F, Ben-Shachar MS. Package afex: analysis of factorial experiments. R package version 0.27-2. 2020.

[ref61] Steinhauser M, Yeung N. Decision processes in human performance monitoring. J Neurosci. 2010:30:15643–15653. 21084620 10.1523/JNEUROSCI.1899-10.2010 PMC3073548

[ref62] Tibshirani R . Regression shrinkage and selection via the lasso. J R Stat Soc Series B Stat Methodology. 1996:58:267–288.

[ref63] Turner BO, Paul EJ, Miller MB, Barbey AK. Small sample sizes reduce the replicability of task-based fMRI studies. Commun Biol. 2018:1:62. 30271944 10.1038/s42003-018-0073-z PMC6123695

[ref64] Ullsperger M, Harsay HA, Wessel JR, Ridderinkhof KR. Conscious perception of errors and its relation to the anterior insula. Brain Struct Funct. 2010:214:629–643. 20512371 10.1007/s00429-010-0261-1 PMC2886909

[ref65] Van Boxtel GJ, Van Der Molen MW, Jennings JR. Differential involvement of the anterior cingulate cortex in performance monitoring during a stop-signal task. J Psychophysiol. 2005:19:1–10.

[ref23] van Gaal S, Ridderinkhof KR, van den Wildenberg WPM, Lamme VAF. Dissociating consciousness from inhibitory control: evidence for unconsciously triggered response inhibition in the stop-signal task. J Exp Psychol Hum Percept Perform. 2009:35:1129–1139. 19653754 10.1037/a0013551

[ref66] van Veen V, Carter CS. Error detection, correction, and prevention in the brain: a brief review of data and theories. Clin EEG Neurosci. 2006:37:330–335. 17073172 10.1177/155005940603700411

[ref67] Wessel JR . Error awareness and the error-related negativity: evaluating the first decade of evidence. Front Hum Neurosci. 2012:6:88. 22529791 10.3389/fnhum.2012.00088 PMC3328124

[ref68] Wessel JR, Danielmeier C, Ullsperger M. Error awareness revisited: accumulation of multimodal evidence from central and autonomic nervous systems. J Cogn Neurosci. 2011:23:3021–3036. 21268673 10.1162/jocn.2011.21635

[ref69] Zigmond AS, Snaith RP. The hospital anxiety and depression scale. Acta Psychiatr Scand. 1983:67:361–370. 6880820 10.1111/j.1600-0447.1983.tb09716.x

