# Supplementary material for: Examining the neural correlates of error awareness in a large fMRI study
Source: Cereb Cortex. 2022 Mar 3;33(2):458–68. doi: 10.1093/cercor/bhac077 (PMC9837605; doi:10.1093/cercor/bhac077)
Supplement: supplementary_material_bhac077 [file supplementary_material_bhac077.docx]

**Supplementary Material**

**Table 1A**

*Number of Participants Per Age Group*

| Age group | *n* |
| --- | --- |
| 18-19 | 97 |
| 20-29 | 258 |
| 30-39 | 38 |
| 40-49 | 8 |
| 50 | 1 |

**Table 2A**

*Summary of Pilot Data*

| No-Go condition | Mean awareness rate (%) | Mean inhibition accuracy (%) |
| --- | --- | --- |
| One-rule task |  |  |
| Colour | 80 | 54 |
| Repeat | 85 | 65 |
| Two-rule task |  |  |
| Colour | 82 | 40 |
| Repeat | 75 | 51 |

*Note. N* = 12

**Table 3A**

*Trial Numbers for Analyses on Reaction Time and Post-No-Go Reaction Time Adjustments*

|  | *M* | *SD* | *n* |
| --- | --- | --- | --- |
| Reaction time |  |  |  |
| Go response | 609.27 | 39.95 | 402 |
| Aware error | 49.37 | 30.31 | 402 |
| Unaware error | 8.58 | 8.38 | 402 |
| Post-No-Go reaction time |  |  |  |
| Correct inhibition | 66.18 | 23.43 | 402 |
| Aware error | 46.38 | 21.54 | 402 |
| Unaware error | 7.14 | 6.86 | 402 |

**Table 4A**

*Descriptive Statistics of Lasso Regression Independent Variables*

|  | *M* | *SD* | *n* |
| --- | --- | --- | --- |
| BIS-11 – attention | 16.93 | 3.43 | 364 |
| BIS-11 – motor | 23.00 | 4.12 | 364 |
| BIS-11 – plan | 24.05 | 4.45 | 364 |
| AQ – social skill | 2.83 | 1.97 | 374 |
| AQ – attention switching | 4.24 | 1.89 | 374 |
| AQ – attention to detail | 4.84 | 2.38 | 374 |
| AQ – communication | 2.71 | 1.93 | 374 |
| AQ – imagination | 2.58 | 1.85 | 374 |
| BIS/BAS – BAS drive | 11.22 | 2.10 | 322 |
| BIS/BAS – BAS fun | 11.79 | 1.96 | 322 |
| BIS/BAS – BAS reward | 16.57 | 2.05 | 322 |
| BIS/BIS – BIS score | 19.23 | 2.30 | 322 |
| HADS – anxiety | 11.07 | 3.12 | 373 |
| HADS – depression | 7.62 | 2.41 | 373 |
| CAARS – attention | 54.33 | 9.09 | 374 |
| CAARS – hyperactivity | 53.84 | 8.57 | 374 |
| CAARS – impulsivity | 50.29 | 9.27 | 374 |
| CAARS – self-concept | 52.76 | 10.31 | 374 |
| CAARS – DSM attention | 53.89 | 14.19 | 374 |
| CAARS – DSM hyperactivity | 50.85 | 10.52 | 372 |
| CAARS – DSM ADHD | 53.28 | 12.22 | 372 |
| CAARS – index | 53.38 | 9.16 | 374 |

*Note.* Norm score ranges: BIS-11 total scores between 52 and 71 (Stanford et al. 2009); AQ total scores below 29 (Broadbent et al. 2013); HADS subscale scores less than 11 (Zigmond and Snaith 1983); CAARS subscale scores of approximately 50 (Conners et al. 1999). The BIS/BAS scale does not have a defined cut-off. BIS, Barratt Impulsiveness Scale, Version 11; AQ, Autism Spectrum Quotient; BIS/BAS, Behavioural Inhibition/Avoidance Scale; HADS, Hospital Anxiety and Depression Scale; CAARS, Conners’ Adult ADHD Rating Scales.

**References**

Broadbent J, Galic I, Stokes MA. 2013. Validation of Autism Spectrum Quotient Adult Version in an Australian Sample. Autism Res. 984205.

Conners CK, Erhardt D, Epstein JN, Parker JDA, Sitarenios G, Sparrow E. 1999. Self-ratings of ADHD symptoms in adults I: Factor structure and normative data. J Atten Disord. 3:141-151.

Stanford MS, Mathias CW, Dougherty DM, Lake SL, Anderson NE, Patton JH. 2009. Fifty years of the Barratt Impulsiveness Scale: An update and review. Pers Individ Differ. 47:385-395.

Zigmond AS, Snaith RP. 1983. The hospital anxiety and depression scale. Acta Psychiatr Scand. 67:361-370.
